# ‘Mito-Bomb’: a novel mitochondria-targeting nanosystem for ferroptosis-boosted sonodynamic antitumor therapy

**DOI:** 10.1080/10717544.2022.2126027

**Published:** 2022-09-21

**Authors:** Jianxin Wang, Zhiyu Zhao, Yan Liu, Xinyu Cao, Fuxin Li, Haitao Ran, Yang Cao, Changjun Wu

**Affiliations:** a Department of Ultrasound, The First Affiliated Hospital of Harbin Medical University, Harbin, China; b Chongqing Key Laboratory of Ultrasound Molecular Imaging, Institute of Ultrasound Imaging, Second Affiliated Hospital, Chongqing Medical University, Chongqing, China

**Keywords:** Sonodynamic therapy, ferroptosis, reactive oxygen species, GPX4, RSL-3

## Abstract

Mitochondria play an important role in regulating tumor cell death and metabolism so that they can be potential therapeutic targets. Sonodynamic therapy (SDT) represents an attractive antitumor method that induces apoptosis by producing highly toxic reactive oxygen species (ROS). Mitochondria-targeting SDT can cause oxidative damage and improve the efficiency of tumor therapy. However, due to the nonselective distribution of nanosystems and the anti-apoptotic mechanism of cancer cells, the therapeutic effect of SDT is not ideal. Therefore, we proposed a novel mitochondria-targeting nanosystem (‘Mito-Bomb’) for ferroptosis-boosted SDT. Sonosensitizer IR780 and ferroptosis activator RSL-3 were both encapsulated in biocompatible poly(lactic‐*co*‐glycolic acid) (PLGA) nanoparticles to form ‘Mito-Bomb’ (named IRP NPs). IR780 in this nanosystem was used to mediate mitochondria-targeting SDT. RSL-3 inhibited the activity of GPX4 in the antioxidant system to induce ferroptosis of tumor cells, which could rewire tumor metabolism and make tumor cells extremely sensitive to SDT-induced apoptosis. Notably, we also found that RSL-3 can inhibit hypoxia inducible factor-1α (HIF-1α) and induce ROS production to improve the efficacy of SDT to synergistically antitumor. RSL-3 was applied as a ‘One-Stone-Three-Birds’ agent for cooperatively enhanced SDT against triple-negative breast cancer. This study presented the first example of RSL-3 boosting mitochondria-targeting SDT as a ferroptosis activator. The ‘Mito-Bomb’ biocompatible nanosystem was expected to become an innovative tumor treatment method and clinical transformation.

## 1. Introduction

Sonodynamic therapy (SDT), as a new treatment strategy for tumor treatment, has overcome the limitation of light penetration depth and has the advantages of minimally invasive, no radiation, and low cost (Lafond et al., 2019; Son et al., 2020; Jiang et al., 2022). Although the detailed mechanism of SDT is still unclear, it is evident that low-intensity ultrasound will lead to excessive reactive oxygen species (ROS) production when interacting with sonosensitizers, thus enhancing cytotoxicity (Son et al., 2020; Zhang et al., 2021b).In addition, an essential factor affecting SDT is the subcellular localization of sonosensitizers. Mitochondria, as the site of cell energy metabolism, also participate in various types of cell death, which has attracted the attention of researchers (Pathania et al., 2009; Guo et al., 2021). Previous studies have shown that destroying mitochondrial integrity can transmit apoptotic cell signals and initiate apoptosis. Mitochondria-targeting SDT resulted in more tumor cell apoptosis than SDT with nonselective subcellular distribution (Zong et al., 2016; Qu et al., 2020; Han et al., 2021). Therefore, mitochondria-targeting SDT is considered a more effective and promising treatment for apoptosis. However, due to the inherent anti-apoptotic ability of triple-negative breast cancer tumor (TNBC) cells, the overall effectiveness of various treatment methods, including SDT and the improvement of patient survival rate, are still not ideal (Sun et al., 2021; Yao et al., 2021). There is an urgent need to develop non-apoptotic therapies to disrupt the anti-apoptotic mechanisms of TNBC cells.

Ferroptosis differs from apoptosis and other types of cell death regulation in morphology and mechanism. Morphologically, it is characterized by the atrophy of mitochondria and a decrease in the number of mitochondrial cristae (Luo et al., 2021). As a cell death mode different from traditional apoptosis, ferroptosis can cause cell death by inhibiting the glutathione-dependent antioxidant defense mechanism and the uncontrolled production of lipid ROS catalyzed by iron ions, mainly the accumulation of lipid peroxidation (LPO) (Stockwell et al., 2017; Hirschhorn & Stockwell, 2019). This can overcome the limitations of existing antitumor therapies and enhance tumor treatment through synergistic effects with SDT. Glutathione peroxidase 4 (GPX4), mainly located in the cytoplasm, can be a core regulator of ferroptosis (Dixon et al., 2012; J. Li et al., 2020). GPX4 combats with LPO by using two glutathione molecules as electron donors to reduce cytotoxic LPO to the corresponding alcohols (Yang et al., 2014; Xu et al., 2021). Inhibiting GPX4 activity can lead to LPO accumulation, a significant feature of ferroptosis (Wei et al., 2020; Tang et al., 2021). In addition, studies have also confirmed that direct targeting of GPX4 is more effective than the destruction of GSH in cancer treatment based on ferroptosis (Shintoku et al., 2017; X. Li et al., 2022). Methyl (1*S*,3*R*)-2-(2-chloroacetyl)-1-(4-(methoxycarbonyl)phenyl)-2,3,4,9-tetrahydro-1*H*-pyrido[3,4-*b*]indole-3-carboxylate (RSL-3) contains electrophilic chloroacetamide moiety, which irreversibly binds to its active site, directly inhibiting the catalytic activity of GPX4 (Eaton et al., 2020). Thus, the redox balance in tumor cells is destroyed, and ferroptosis is induced.

**Scheme 1.**
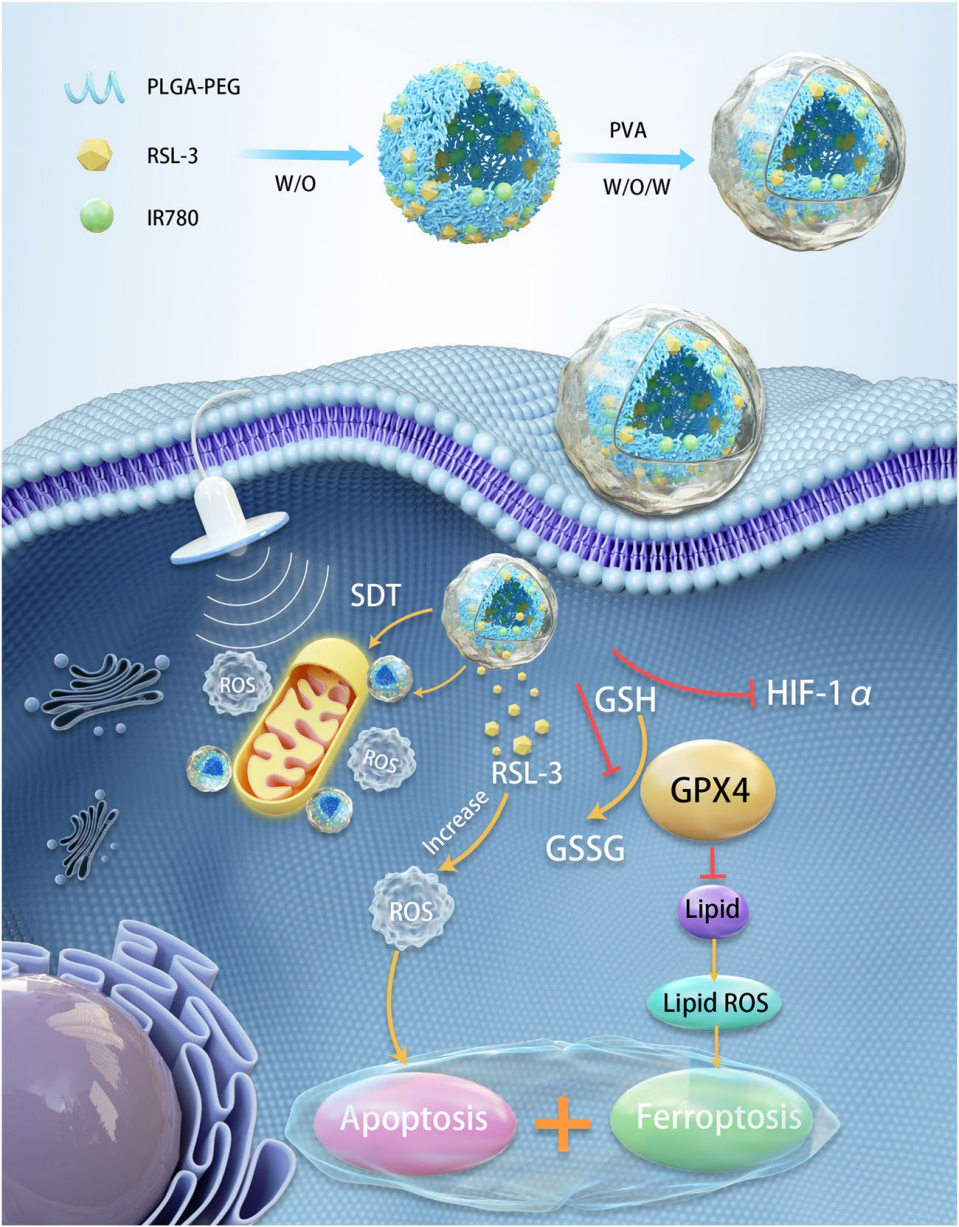


Capitalizing on these facts, we innovatively propose a novel mitochondria-targeting nanosystem (‘Mito-Bomb’) for ferroptosis-boosted SDT. We used FDA-approved degradable poly(lactic‐*co*‐glycolic acid) (PLGA) to encapsulate oil-soluble drugs IR780 and RSL-3 (Scheme 1). The problem of low solubility and poor pharmacokinetics of two drugs was solved by using PLGA as the carrier to improve the possibility of its clinical application. IR780 has been confirmed to accumulate in mitochondria preferentially (Wang et al., 2013; Zhang et al., 2018). IR780 mediates mitochondrial-targeted SDT and can perform visual and accurate treatment under photoacoustic imaging (PAI) guidance. PAI is a new noninvasive and non-ionizing biomedical imaging method developed in recent years, which can monitor the treatment process in real time and visualize tumor treatment (Steinberg et al., 2019; Glickman, 2021). RSL-3 as a ‘One-Stone-Three-Birds’ agent for cooperatively enhanced SDT against breast cancer. RSL-3 has confirmed its sensitization effect on SDT in vitro and in vivo by simultaneously inhibiting GPX4 and hypoxia inducible factor-1α (HIF-1α) and promoting ROS production. The mitochondria-targeting nanosystem (‘Mito-Bomb’) can simultaneously ferroptosis-boosted SDT against TNBC. This nanosystem can accumulate into the tumor through intravenous injection and significantly inhibit tumor growth without any adverse side effects, which provides an avenue for therapeutic intervention against TNBC in the clinic.

## 2. Material and methods

### 2.1. Materials and reagents

PEGylated PLGA (50:50, PLGA 12,000 Da MW, PEG 2,000 Da MW) (PLGA-PEG_2,000_) was purchased from Xi’an Ruixi Biological Technology Co., Ltd. (Shaanxi, China). IR780 iodide,1,1-dioctadecyl-3,3,3′,3′-tetramethylindocarbocyanine perchlorate (DiI), 2′-7′-dichlorofluorescein diacetate (DCFH-DA), and polyvinyl alcohol (PVA) were purchased from Sigma-Aldrich (St Louis, USA). RSL-3 was purchased from MedChemExpress (Shanghai, P.R. China). Anti-Caspase-3, anti-HIF-1α, and anti-GPX4 antibodies were purchased from Abcam (London, U.K.). 5,5′,6,6′-Tetrachloro-1,1′,3,3′-tetraethyl-imidacarbocyanine iodide (JC-1) assay kit and 4,6-diamidino-2-phenylindole (DAPI) were from Beyotime Biotechnology (Shanghai, China). BODIPY-581/591C11 reagent, MitoTracker, and Singlet Oxygen Sensor Green (SOSG) were purchased from Thermo Fisher Scientific Co., Ltd. (Carlsbad, California, UK). Cell Counting Kit-8 (CCK-8) assays were obtained from Dojindo (Kumamoto, Japan).

### 2.2. Synthesis of IRP NPs

Briefly, 50 mg of PLGA-PEG_2,000_, 2 mg of IR780, and 1 mg of RSL-3 were dissolved in 2 mL of dichloromethane (CH_2_Cl_2_). Briefly, 200 μL of ultra-pure water was added to the above oil phase mixture using the probe sonicator (Sonics & Materials Inc., USA) at an intensity of 60 W for 3 min. Subsequently, 5 mL of 4% PVA aqueous solution was added to the above solution for the second emulsification at an intensity of 45 W for 3 min. About 10 mL of isopropanol solution (2%) was added and magnetically stirred for 3 h to remove CH_2_Cl_2_. Finally, the IRP NPs were purified by centrifugation (11,000 rpm, 6 min). The PLGA encapsulated IR780, and RSL-3 nanoparticles were named IRP NPS. The same operation process was also applicable to the synthesis of IP NPs (PLGA encapsulated IR780 nanoparticles), except that RSL-3 was not added.

### 2.3. Characterization of IRP NPs

The internal structure of IRP NPs was observed by transmission electron microscopic (TEM, FEI Tecnai G2 F20, USA). The size and zeta potential were determined using dynamic light scattering (DLS, Malvern Instruments Ltd., UK). The UV–vis absorption spectra of PLGA, free IR780, IP NPs, and IRP NPs were obtained using a UV–vis spectrophotometer (US-2550, Shimadzu, Japan). A calibration curve was drawn using absorbance obtained at different concentrations of IR780. The encapsulation efficiency of IR780 was calculated using the standard curve. The encapsulation efficiency of RSL-3 was also measured by high-performance liquid chromatography (HPLC; ShmadzulC-2-1-AHT; Japan). To obtain the release profiles of IR780 and RSL-3, we dispersed IRP NPs into phosphate buffered saline (PBS) and transferred the mixture to a dialysis membrane. Then, the membrane was put into a glass bottle containing PBS. After shaking at 37 °C for 2 h, the solution was divided into two groups. One group was irradiated with LIFU, and the other served as a control. Obtained 1 mL of solution at predetermined time points (0, 1, 2, 4, 8, 12, 18, and 24 h), and then added 1 mL of PBS to the bottle. UV–vis spectrophotometer and HPLC, respectively, were used to measure the concentrations of IR780 and RSL-3. ROS(^1^O_2_) production was assessed in vitro using fluorescence spectroscopy (RF-5301PC, Shimadzu, Japan) of SOSG (*λ*_ex_/*λ*_em_ = 504 nm/525 nm).

### 2.4. Cellular uptake and distribution

The murine TNBC cell line 4T1 was obtained from Chongqing Key Laboratory of Ultrasound Molecular Imaging and cultured in RPMI-1640 medium containing 10% fetal bovine serum and 1% penicillin–streptomycin at 37 °C and 5% CO_2_. 4T1 cells were seeded into glass-bottom cell culture dishes (1 × 10^5^ cells per dish) and incubated overnight. DiI-labeled IRP NPs (1 mL, 50 μg/mL) were co-incubated with 4T1 cells for different time points (0.5, 1, 2 and, 4 h). Then, the dishes were rinsed with PBS three times. After fixation with 4% paraformaldehyde, 200 μL of DAPI was added to each dish (8 min). Then, cellular uptake was observed by the Laser Confocal Scanning Microscopic (CLSM, Nikon A1, Japan). To quantify intracellular uptake, 4T1 cells were seeded in 6-well plates (1 × 10^5^ cells per well) and incubated overnight to reach 80%–90% confluency. DiI-labeled IRP NPs (1 mL, 50 μg/mL) were co-incubated with 4T1 cells for different durations (0.5, 1, 2, and 4 h). Cells were digested and collected, then measured intracellular red fluorescence by flow cytometry (BD facvantage se, USA).

To ascertain the mitochondria-targeting ability of IRP NPs, 4T1 cells (1 × 10^5^ cells per dish) were incubated in CLSM dishes overnight. After incubating cells with 50 μg/mL of DiI-labeled IRP NPs for 4 h, cells were rinsed with PBS and dyed with MitoTracker for 30 min to label mitochondria. The mitochondrial localization of IRP NPs was confirmed using CLSM. Furthermore, the Pearson correlation coefficient was measured. To evaluate the co-localization of nanoparticles and lysosomes, MitoTracker was replaced with LysoTracker after the corresponding treatment of cells.

### 2.5. In vitro ROS production detection

ROS production in cells was detected using the green fluorescent probe DCFH-DA (*λ*_ex_/*λ*_em_ = 488 nm/530 nm). 4T1 cells were seeded in CLSM dishes (1 × 10^5^ cells per dish) and randomly distributed into six groups: Control, US, IP NPs, IRP NPs, IP NPs + US, and IRP NPs + US. The concentration of IR780 in each group was 4 μg/mL. After corresponding treatments, the cells were incubated with DCFH-DA (10 μM) in each dish and imaged with CLSM after three times of PBS washing. The collected cells were subjected to ROS quantification analysis by flow cytometry.

### 2.6. Mitochondrial membrane potential assay

4T1 cells were seeded in CLSM dishes (1 × 10^5^ cells per dish) and incubated overnight. Next, dishes were randomly divided into the above six groups and treated accordingly. The concentration of IR780 in each group was 4 μg/mL. Dishes were stained with JC-1 following the manufacturer’s protocol. Cells treated as aforementioned were collected for flow cytometry.

### 2.7. Cell viability study and apoptosis assay

The following six groups were performed after overnight incubation. Add CCK-8 reagent to the cell culture medium of each group, measure the absorbance at 450 nm, and calculate the cell viability of each group of treatments. Apoptosis was detected by flow cytometry after double staining with Calcein-AM/propidium iodide.

### 2.8. Western blot analysis

The treated proteins in each group were subjected to gel electrophoresis, transfer, and blocking. Incubated the protein with anti-Caspase-3, anti-HIF-1α, and anti-GPX4 antibodies overnight at 4 °C. Horseradish peroxidase-conjugated rabbit IgG secondary antibody was incubated for 1 h at room temperature on a shaker. Visualized and analyzed the protein bands.

### 2.9. LPO Accumulation

4T1 cells were seeded in CLSM dishes (1 × 10^5^ cells per dish) and incubated overnight. After six different treatments, BODIPY-581/591C11 (5 μM, 20 min) was added to each of the different dishes for LPO staining. The dishes were rinsed three times with PBS and LPO accumulation was observed by CLSM and flow cytometry analysis.

### 2.10. PAI of IRP NPs and in vivo biodistribution

For in vitro, PAI (excitation wavelength: 780 nm) was performed on a series of concentrations of IRP NPs at 31.25, 62.5, 125, 250, and 500 μg/mL, and a calibration curve was drawn between the PA values and the concentrations. For in vivo PAI, tumor-bearing mice were injected intravenously with IRP NPs in PBS (10 mg/mL, 200 µL). Intratumoral PAI images were acquired at 0, 1, 3, 6, 12, 24, and 48 h, respectively. The signal intensity within the tumor was analyzed to determine the optimal enrichment time for IRP NPs. The fluorescence characteristics of IR780 endow IRP NPs with fluorescence imaging performance (*λ*_ex_/*λ*_em_ = 745 nm/820 nm), which can further evaluate the biodistribution of IRP NPs. Fluorescence imaging was performed on mice at different times after tail vein injection of IRP NPs.

### 2.11. Antitumor therapy efficacy in vivo

A subcutaneous xenograft model was established by subcutaneous injection of 4T1 cells in BALB/c mice and 30 tumor-bearing mice were randomly divided into the following six groups: Control, US, IP NPs, IRP NPs, IP NPs + US, and IRP NPs + US. Twenty-four hours later, the different materials were injected through the tail vein of mice (10 mg/mL, 200 μL). We irradiated the tumor region of the mice with LIFU (on 2 min, off 2 min, two cycles) at 3 W/cm^2^. Repeat the same operation on the third day. During the 14-day treatment period, mice’s body weight and tumor volume were recorded every two days. After the treatment, tumors and major organs were collected for hematoxylin and eosin (H&E) and TdT-dependent dUTP-biotin nick end labeling (TUNEL) and HIF-1α staining.

### 2.12. Biosafety of IRP NPs

Using healthy mice as an animal model for the biosafety of IRP NPs, we injected IRP NPs intravenously (10 mg/mL, 200 μL). Mice were sacrificed at specific time points (1, 7, and 14 days) after intravenous injection of IRP NPs, blood was collected for blood routine and biochemical tests, and major organs were collected for H&E staining.

### 2.13. Statistical analysis

All experiments were repeated at least three times, and the data are shown as mean ± standard deviation. The Student’s *t* tests determined statistical comparisons between groups (*p < .05, **p < .01, ***p < .001, ****p < .0001).

## 3. Results and discussion

### 3.1. Design, synthesis, and characterization of IRP NPs

**Figure 1.**
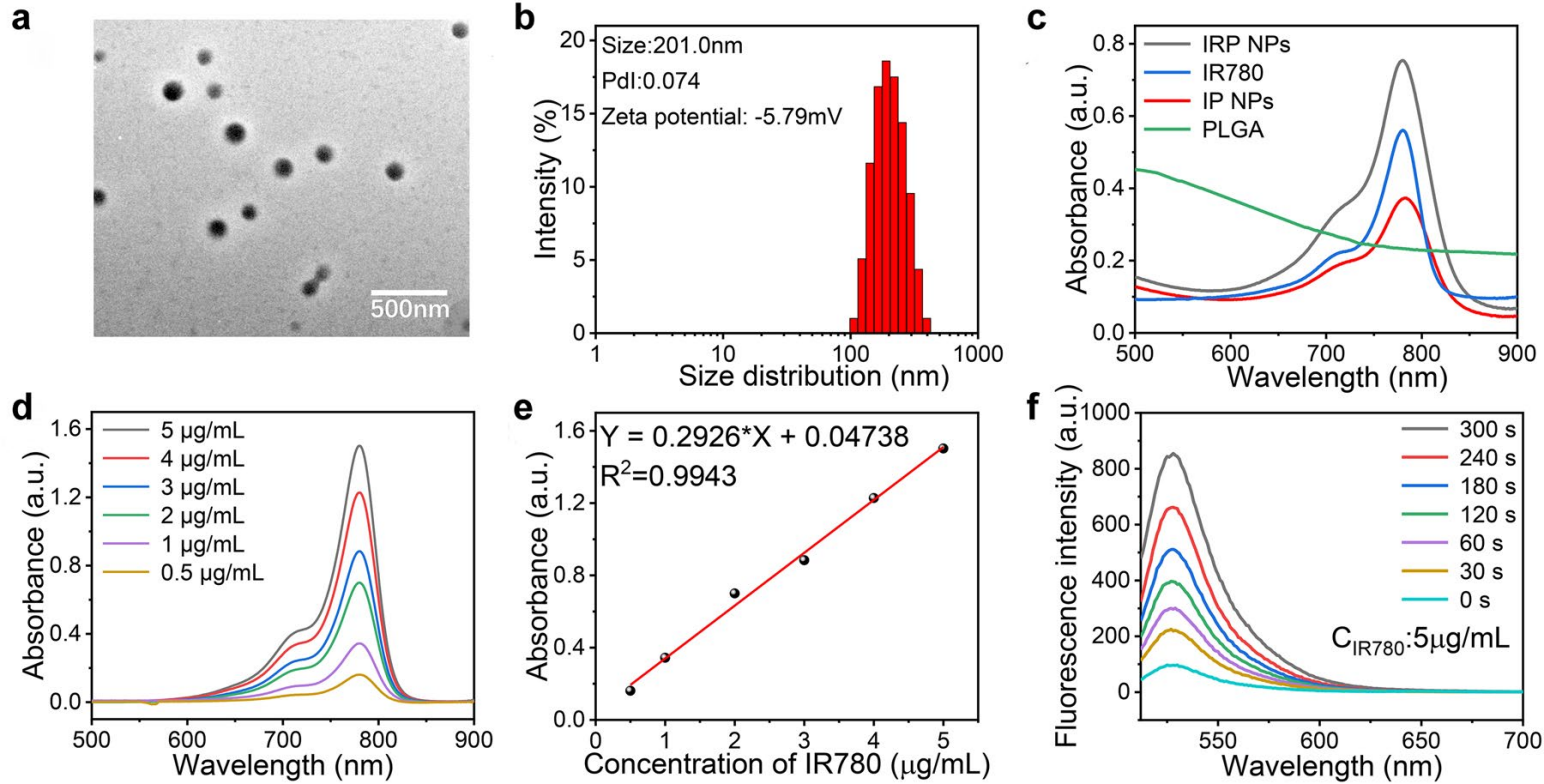
Characterizations of IRP NPs. (a) TEM of IRP NPs. (b) Size distribution, PdI, and Zeta potential of IRP NPs. (c) Absorbance spectra of different NPs (PLGA, Free IR780, IP, IRP) as recorded by UV–vis spectra. (d) UV–vis absorbance spectra of free IR780 in different concentrations. (e) The standard curve of IR780 and UV–vis absorbance. (f) Time-dependent ^1^O_2_ generation of IRP NPs irradiated by US.

IRP NPs were synthesized through a typically double-emulsion approach (Deng et al., 2017), in which IR780 and RSL-3 were stably embedded in the PLGA shell. TEM characterization revealed that IRP NPs have a uniform spherical structure (Figure 1(a)). The average size of IRP NPs measured by a DLS system was 201.0 nm, consistent with the TEM results (Figure 1(b)). The zeta potential result exhibited an average potential of –5.49 mV (Figure 1(b)). The UV–vis spectrum of IR780 in different concentrations of dichloromethane showed a typical absorption peak near 780 nm. Both IRP NPs and IP NPs showed the characteristic absorption of free IR780, implying that the drug was successfully loaded (Figure 1(c)). The UV–vis spectrum absorption intensity of IR780 is concentration-dependent and has a good linear correlation shows a concentration (Figure 1(d,e)). The encapsulation rate of IR780 in IP NPs and IRP NPs was calculated to be 80.67% and 74.7%, respectively. The encapsulation rate of RSL-3 IRP NPs was 68.81%, obtained by HPLC. Compared with the irradiation group without LIFU, LIFU irradiation caused the rapid release of IR780 and RSL-3, which may be due to the rapid and enhanced release of drugs caused by the oscillation and cavitation effects caused by LIFU (Supplementary Figure S1 and S2). To study the efficiency of SDT, the generated ROS was measured by SOSG. IRP NPs have the ability to produce ^1^O_2_ under US irradiation, and the fluorescence intensity increases with the irradiation time, which laid the foundation for the later SDT treatment (Figure 1(f)).

**Figure 2.**
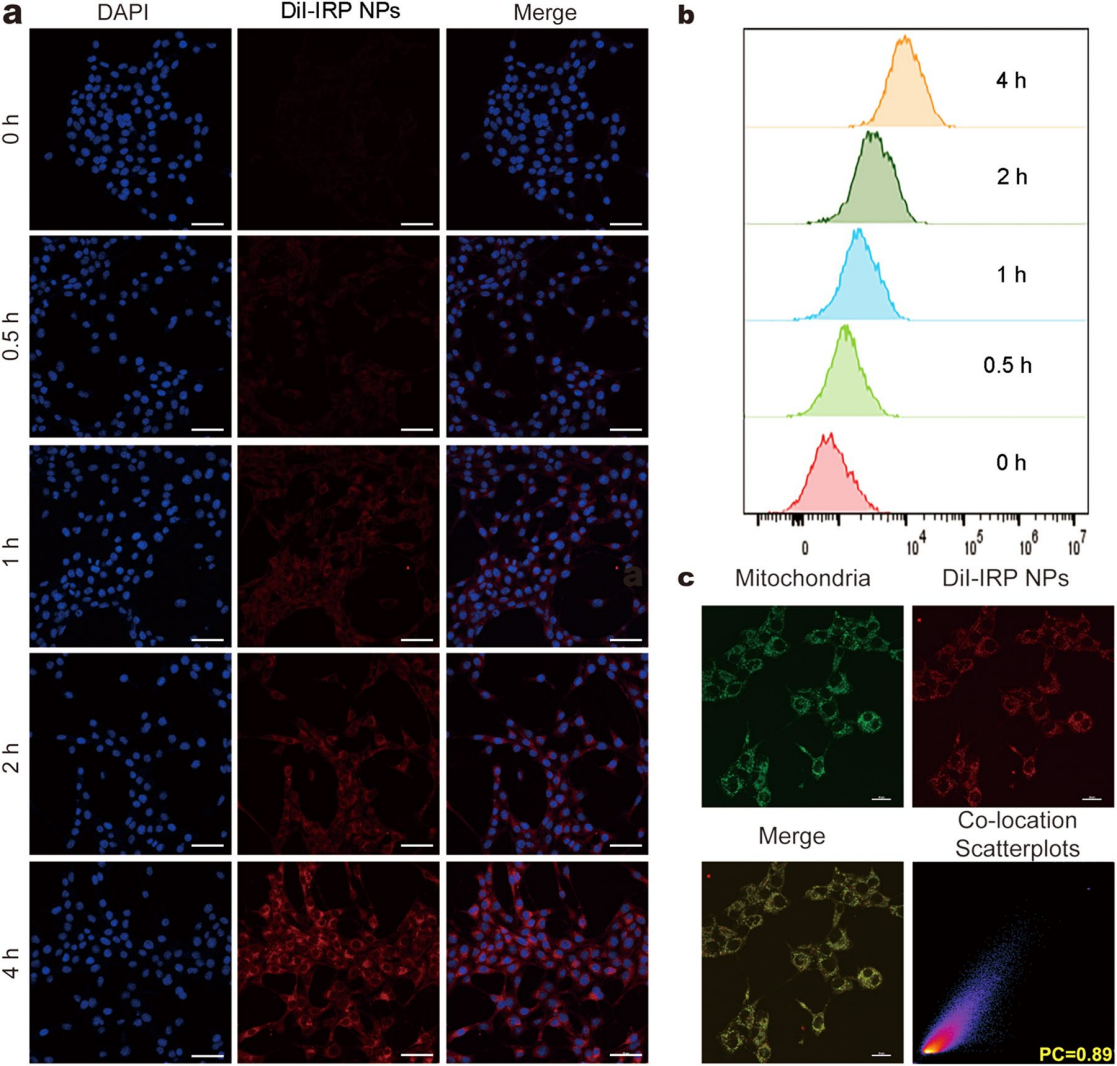
Cellular uptake behaviors and subcellular accumulation of IRP NPs. (a) Cellular uptake of IRP NPs observed using CLSM (scale bar: 50 µm). (b) Cellular uptake of DiI-labeled IRP NPs by flow cytometry analysis. (c) Mitochondrial location of DiI-labeled IRP NPs as monitored by MitoTracker (scale bar: 20 µm).

### 3.2. Cellular uptake and subcellular accumulation

The uptake of nanoparticles by tumor cells is the basis for subsequent therapy (Adjei et al., 2014). To assess the cellular uptake of IRP NPs by 4T1 cells, we labeled IRP NPs with red fluorescent dye DiI and incubated them with cells at different times. The CLSM and flow cytometric analysis showed that IRP NPs could be internalized by cells in a time-dependent manner (Figure 2(a,b)). That is to say, with the prolongation of incubation time, the uptake of IRP NPs in 4T1 cells gradually increased.

The subcellular localization of nanomaterials can significantly affect the therapeutic efficiency of tumors (Liew et al., 2021). Due to the inherent lipophilicity and overall positive charge of IR780, these compounds usually accumulate in mitochondria (Zhang et al., 2010). In order to study this result, after the DiI-labeled NPs were incubated with MitoTracker, the subcellular localization was studied by CLSM, and the intracellular luminescence patterns were compared. The red fluorescence of DiI-labeled NPs overlapped well with the green fluorescence of MitoTracker. The co-localization coefficient was 0.89, indicating that IRP NPs had mitochondrial targeting (Figure 2(c)). Comparatively, the poor overlap was observed in the case of LysoTracker (Supplementary Figure S3).

**Figure 3.**
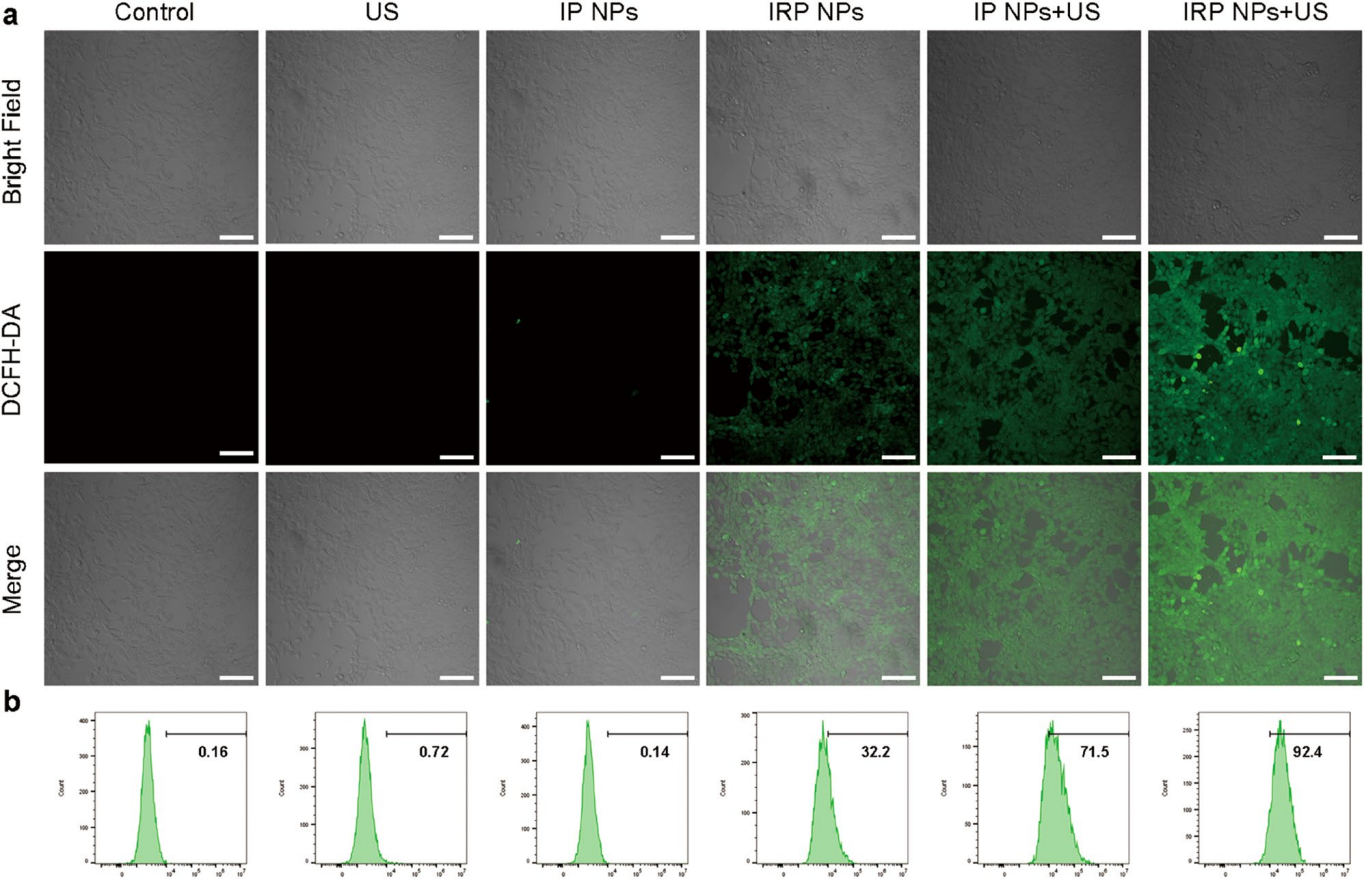
Intracellular ROS generation of IRP NPs. (a) The intracellular ROS levels were determined by DCFH-DA probe reagent and imaged by CLSM images of 4T1 cells (scale bar: 100 µm). (b) The intracellular ROS levels were stained with DCFH-DA probe reagent and detected by flow cytometry.

### 3.3. In vitro SDT and ferroptosis effects of IRP NPs

#### 3.3.1. In vitro ROS production detection

Studies have shown that ROS production is tightly associated with SDT and ferroptosis. ROS triggers substantial modification of biomolecules at the cellular level, thus activating the apoptosis pathway (Su et al., 2019). The intracellular ROS generation capability of IRP NPs was detected by a ROS probe (DCFH-DA). According to Figure 3(a), cells treated with IRP NPs and IP NPs + US groups showed similarly strong green fluorescence and the most ROS in IRP NPs + US group. The results of flow cytometry showed that 4T1 cells treated with IRP NPs exhibited significant ROS compared to the control group (32.2%, Figure 3(b)). This also means RSL-3 could promote ROS production in 4T1 cells. The most vigorous green fluorescence intensity in the IRP NPs + US group (92.4%) was markedly higher than IP NPs (71.5%). The above results confirm that both RSL-3 and SDT can generate ROS, and the two have a significant synergistic effect.

#### 3.3.2. Mitochondrial membrane potential assay

**Figure 4.**
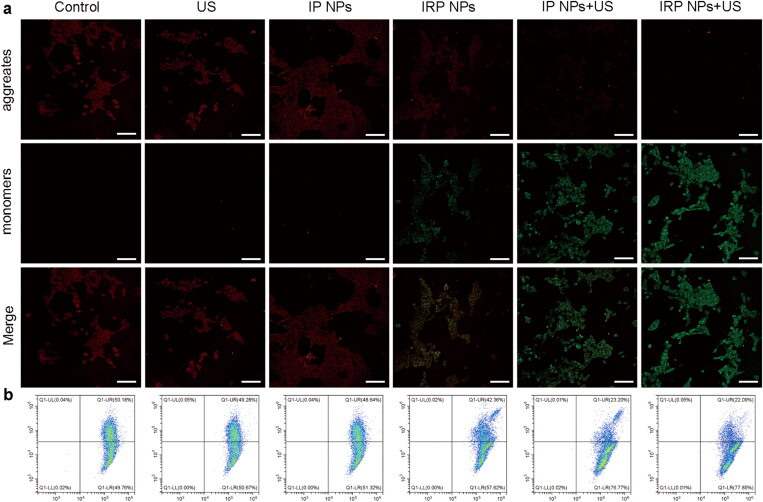
Mitochondria membrane potential. (a) Mitochondria membrane potential changes of 4T1 cells after corresponding treatments observed using CLSM (scale bar: 100 µm). (b) JC-1 assay as a measure of mitochondrial depolarization by flow cytometry.

Selective subcellular accumulation of IRP NPs in the mitochondria of 4T1 cells has been previously validated. Then, the effects of IRP NPs mediated mitochondrial-targeted SDT, and ferroptosis on mitochondrial membrane potential were tested with the JC-1 probe. The cells in the IRP NPs combined with the US irradiation group experienced mitochondrial membrane potential decreased significantly and showed enhanced green and weak red fluorescence (Figure 4(a)). The quantitative results of JC-1 aggregates and JC-1 monomers were measured by flow cytometry. The degree of mitochondrial depolarization was calculated as the red to green fluorescence intensity ratio (Figure 4(b)). The results are consistent with CLSM. These results confirmed that IRP NPs combined with US irradiation reduced mitochondrial membrane potential and disrupted mitochondrial membrane integrity to synergize SDT against TNBC.

#### 3.3.3. Cell viability study and apoptosis assay

**Figure 5.**
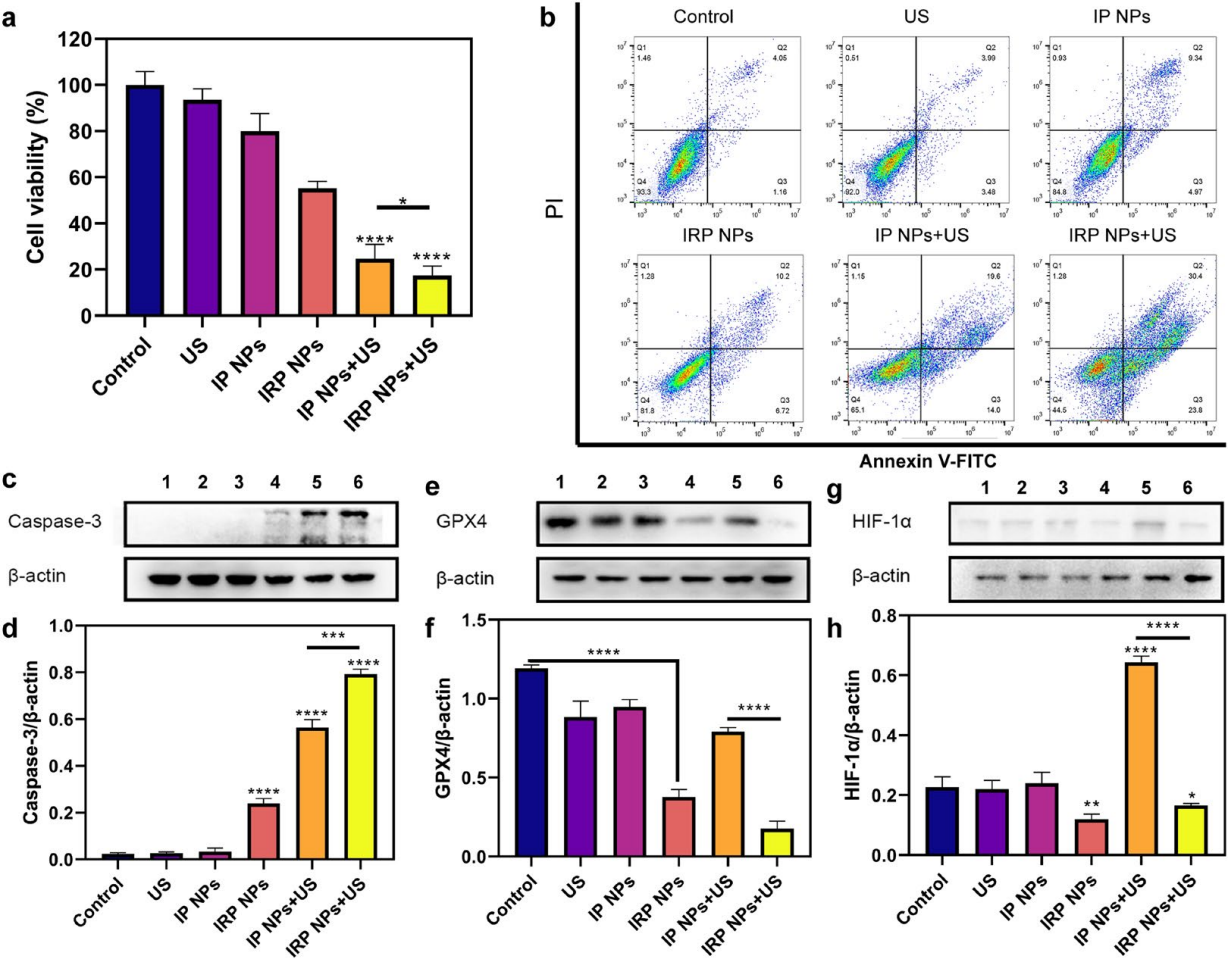
In vitro SDT and ferroptosis effects of IRP NPs. (a) Cell viability of 4T1 cells after different treatments was tested via CCK-8 assay. (b) The apoptosis of 4T1 cells with different treatments was detected by flow cytometry. (c and d) Western blot analysis of Caspase-3 expression in 4T1 cells after the corresponding treatments and quantitative analysis of the Caspase-3 expression. (e and f) Western blot analysis of GPX4 expression in 4T1 cells after the different treatments and quantitative analysis of the GPX4 expression. (g and h) Western blot analysis of HIF-1α expression in 4T1 cells after the different treatments and quantitative analysis of the HIF-1α expression.

After witnessing the enhanced SDT effect after using RSL-3, we then evaluated the cytotoxicity of IRP NPs on 4T1 cells in vitro by CCK-8 assay and apoptosis assay. The CCK-8 assay shown in Figure 5(a) shows that the IP NPs group and IRP NPs showed a slight decrease in cell viability. The cell viability of IRP NPs + US group decreased more significantly than that of IP NPs + US group. The results showed that synergistic therapy was more effective than monotherapy in cytotoxicity.

Next, Annexin V-FITC and PI staining assay was harnessed to detect further the apoptosis of 4T1 cells treated with different methods by flow cytometry (Figure 5(b)). IRP NPs + US group induces 54.2% of cell apoptosis, which distinctly exceeds the IP NPs + US group (33.6%). In conclusion, the results consistently show that IRP NPs have the most effective cytotoxicity, and RSL-3 can enhance the therapeutic effect of SDT.

#### 3.3.4. Western blot analysis

Furthermore, as shown in Figure 5(c,d), we detected the expression of the caspase-3 protein (a typical apoptotic signal) by western blot (WB). We found that the expression of Caspase-3 in SDT combined with the ferroptosis group loaded with RSL-3 was significantly higher than in the SDT group.

Next, we explored the possible mechanism of RSL-3 synergizing SDT. GSH and GPX4 are the central antioxidant systems in vivo, and GPX4 is the glutathione-dependent lipid repair enzyme. When SDT mediates ROS production, RSL-3 can inactivate GPX4 and increase intracellular LPO levels, which is an essential indicator of ferroptosis. Next, WB was used to verify the expression of GPX4 (Figure 5(e,f)). When RSL-3 was present, the expression of GPX4 decreased, and that of the IRP NPs + US group decreased significantly.

In addition, SDT-induced ROS production exacerbates tumor hypoxia, which increases HIF-1α levels and induces increased vascular endothelial growth factor expression, promoting tumor growth and metastasis (Kim et al., 2016; Goyette et al., 2021). Therefore, we also used WB to study the effect of RSL-3 on HIF-1α Expression (Figure 5(g,h)). The results showed that SDT increased the expression of HIF-1α, whereas RSL-3 inhibited the upregulation of HIF-1α expression. This suggests that RSL-3 can alleviate tumor hypoxia and enhance treatment.

#### 3.3.5. LPO accumulation

**Figure 6.**
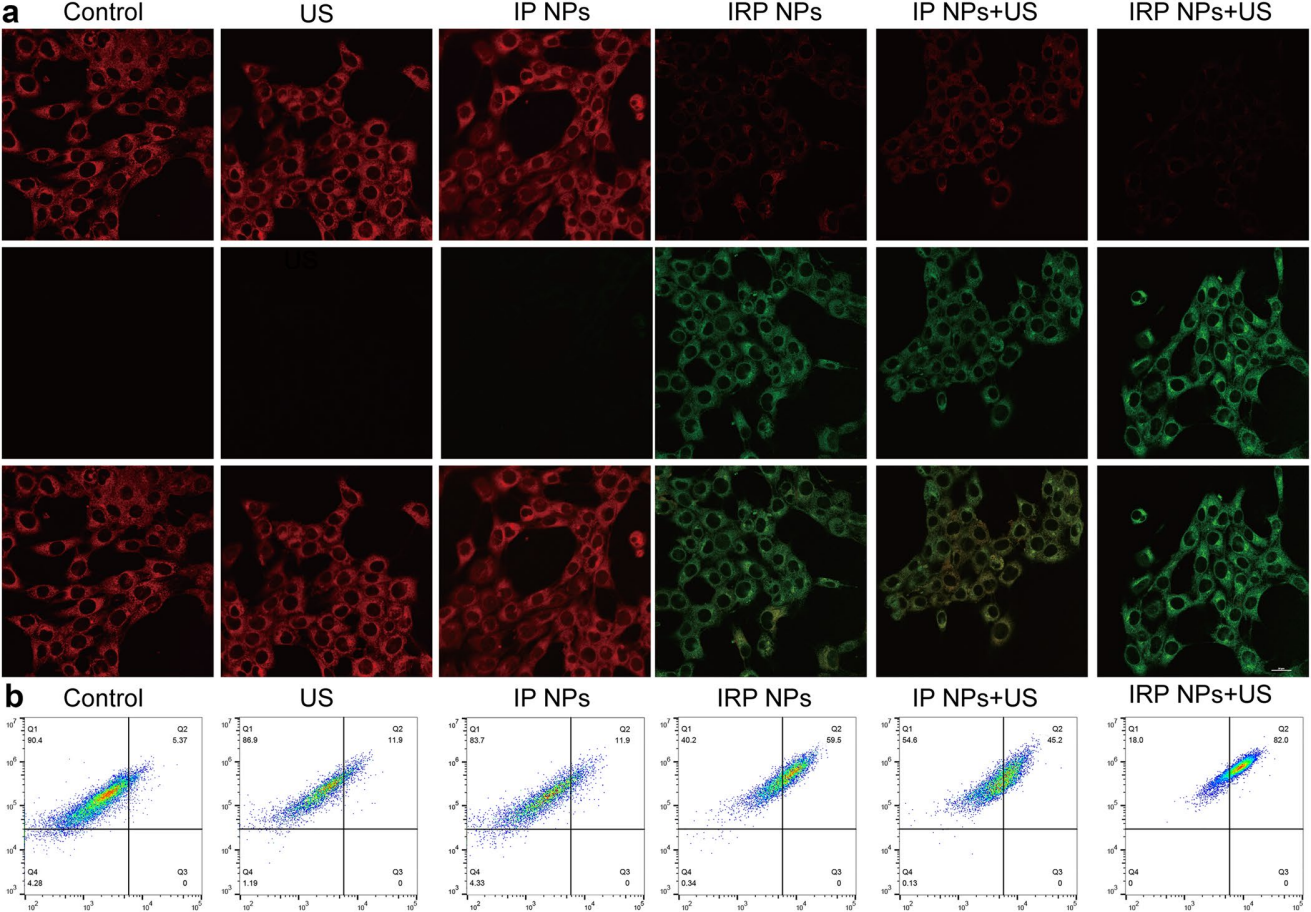
LPO accumulation. (a) LPO accumulation of cells after different treatments observed by CLSM (scale bar: 20 µm). (b) Flow-cytometry-based LPO accumulation assay.

A fundamentally different marker of ferroptosis is the presence of LPO, whose accumulation contributes to the occurrence of ferroptosis (Ursini & Maiorino, 2020). To study whether LPO was produced in cancer cells during treatment, 4T1 cells were incubated with fluorescent LPO-specific dye BODIPY-581/591C11 reagent after various treatments. The results showed that the polyunsaturated butadiene in BODIPY-581/591C11 reagent was partially oxidized by lipid reactive oxygen species (lipid ROS), and the maximum excitation wavelength shifted from 590 nm to 510 nm. The probe still maintained lipophilicity. It can show the level of intracellular lipid ROS. CLSM showed red weakening, and green enhancement (Figure 6(a)). IRP NPs + US group showed the weakest red and the strongest red light, indicating the most LPO accumulation. The amount of LPO was further confirmed by flow cytometry (Figure 6(b)). These results showed that the levels of apoptosis and ferroptosis were the highest in the IRP NPs + US group, which synergistically treated tumors.

### 3.4. PAI of IRP NPs

**Figure 7.**
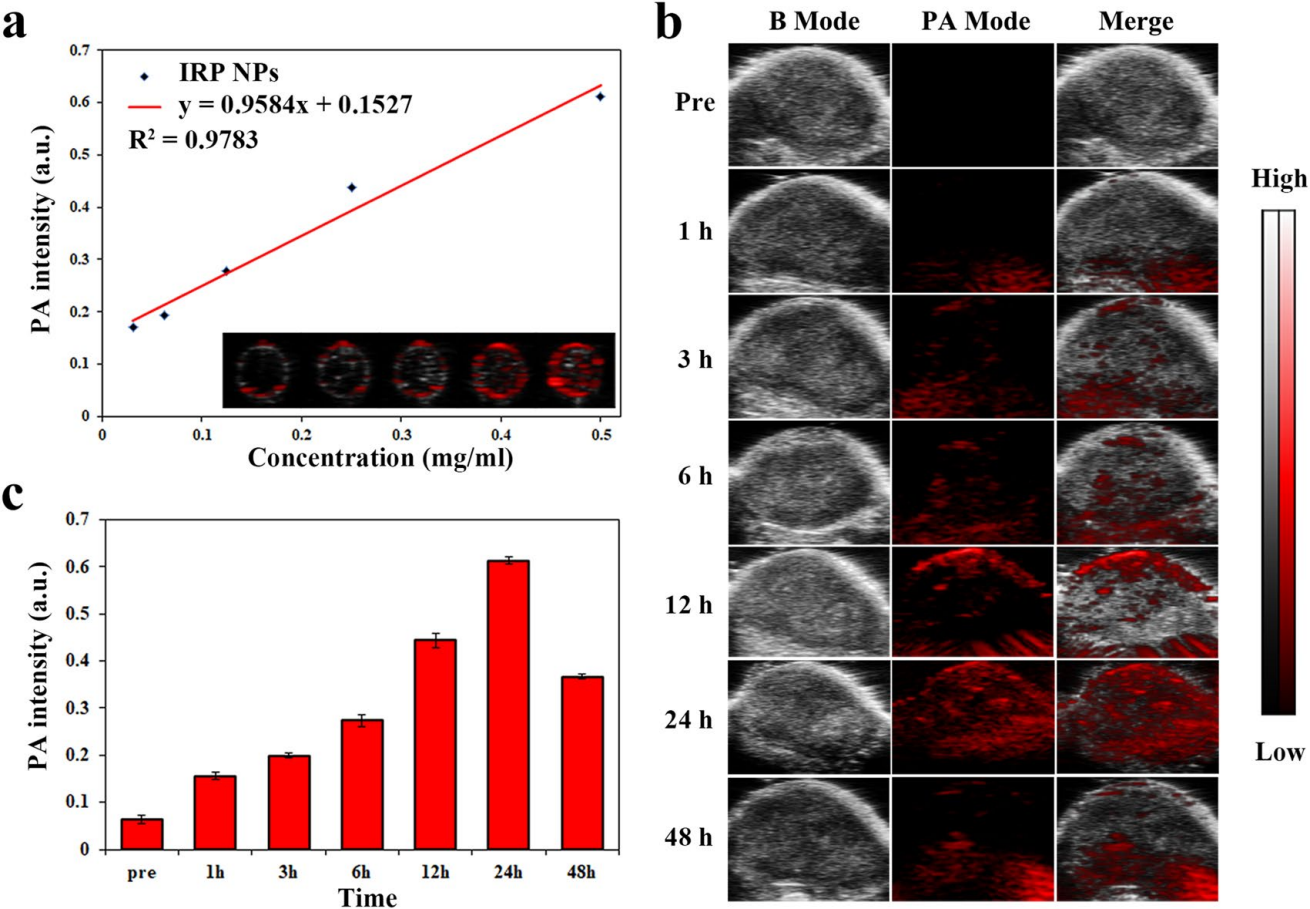
PAI of IRP NPs. (a) The standard curve of PA signal intensity and the concentration of IRP NPs (31.25, 62.5, 125, 250, and 500 μg/mL). (b) PAI of tumors injection of IRP NPs at different time points. (c) The PA signal intensity of IRP NPs in tumor region at different time points.

PA imaging has practical significance in monitoring the accumulation of IRP NPs in tumor areas and guiding tumor treatment in real time. IR780 can be used as a PA contrast agent. We used VEVO LAZR photoacoustic imaging system to perform PA imaging on IRP NPs and 780 nm as excitation wavelength for PAI. As shown in Figure 5(a), the signal intensity of IRP NPs has a clear concentration dependence, which increases from 0.171 to 0.611 with the concentration of IRP NPs (31.25, 62.5, 125, 250, and 500 μg/mL). We then tested the imaging capabilities of IRP NPs in a xenograft model. IRP NPs were injected into the caudal vein to verify whether they could accumulate in the tumor area. The PA signal in the tumor area was observed after 1 h and peaked after 24 h of injection (Figure 7(b,c)). We can verify by PAI that IRP NPs can be enriched within the tumor region. PAI can help evaluate the time of ultrasound intervention and guide the visual treatment process. In vivo fluorescence imaging showed that after injection of IRP NPs, IRP NPs first aggregated in the liver, transferred to the spleen and tumor over time, and finally enriched in the tumor and metabolically attenuated (Supplementary Figure S4).

### 3.5. In vivo antitumor capacity

**Figure 8.**
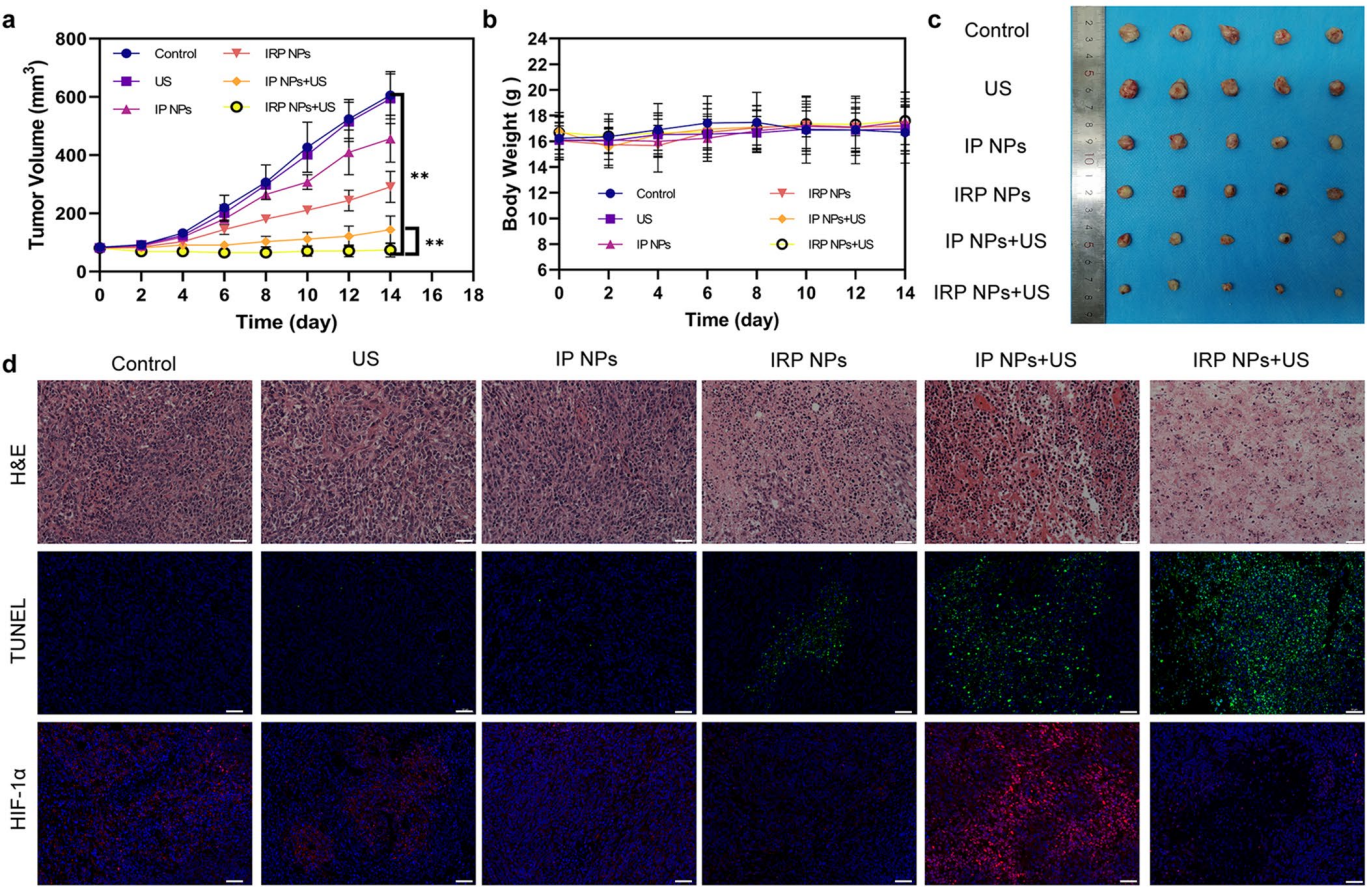
In vivo antitumor effect of IRP NPs. (a) Time-dependent tumor volume curves after different treatments (*n* = 5). (b) Time-dependent body weights curves after different treatments (*n* = 5). (c) Photographs of tumors in each group after treatments. (d) H&E, TUNEL, and HIF-1α staining of tumor regions after different treatments (scale bar: 50 µm).

IRP NPs show the potential to overcome the obstacles of tumor treatment. Next, we will study the in vivo anticancer activity. Next, thirty 4T1 tumor-bearing mice were randomly divided into six groups (control, US, IP NPs, IRP NPs, IP NPs + US, and IRP NPs). The tumor volume of mice and body weight were monitored every two days to plot the growth curve. The control group and US group increased rapidly during the whole treatment period, illustrating that the use of PBS and US alone did not affect limiting tumor growth (Figure 8(a)). The tumor growth of the IP NPs + US group was significantly inhibited, which was related to a large amount of ROS produced during SDT. The tumor growth trend of the IRP NPs + US group was further inhibited. This fantastic therapeutic effect can be attributed to the synergistic effect of RSL-3-mediated ferroptosis and SDT-mediated apoptosis. No significant weight loss or difference was found during treatment (Figure 8(b)). After 14 days of treatment, the mice tumor was removed, and digital photos were taken (Figure 8(c)).

Next, we stained the treated tumors in each group for H&E and TUNEL staining (Figure 8(d)). It was found that severe cell damage occurred in the IRP NPs + US group, which proved the effectiveness of synergistic therapy. After confirming the efficacy, we also measured HIF-1α in tumors (Figure 8(d)). To study the role of RSL-3 in enhancing SDT. It can be seen from the immunofluorescence staining image that SDT can induce the upregulation of HIF-1α (Wang et al., 2018; Zhang et al., 2021a). This may further increase the hypoxia of the tumor. Therefore, RSL-3 can enhance the therapeutic effect of SDT by inhibiting HIF-1α. The above results confirmed the therapeutic advantage of SDT combined with ferroptosis. IRP NPs + US group can effectively induce ferroptosis and increase the susceptibility of 4T1 cells to SDT and induce more cells to undergo apoptosis.

**Figure 9.**
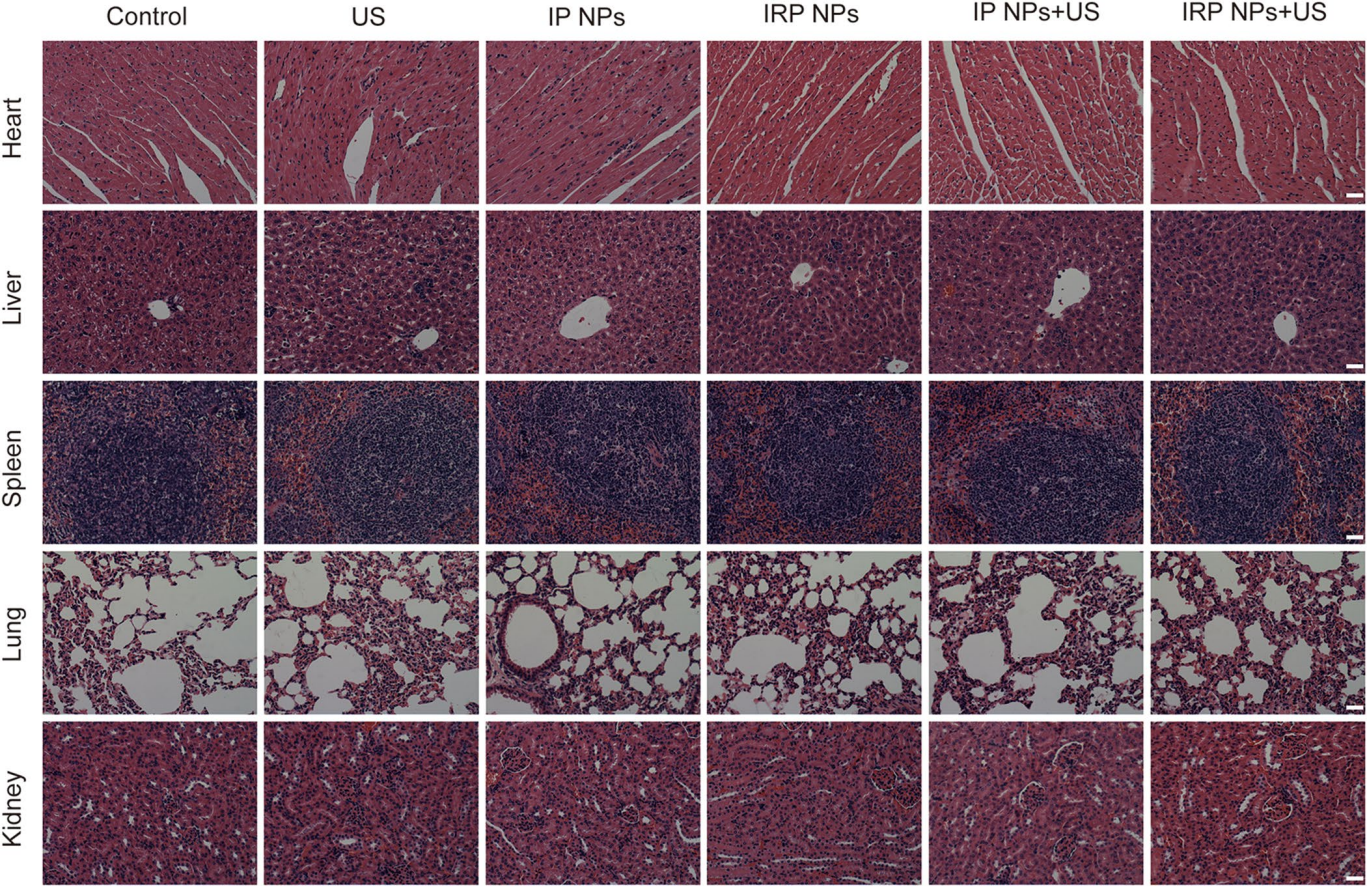
H&E staining of heart, liver, spleen, lung, and kidney after different treatments (scale bar: 50 µm).

Moreover, H&E staining was performed on major organs, including the mice’s heart, liver, spleen, lung, and kidney after treatment (Figure 9). Compared to the control group, no significant histopathological necrosis, hydropic degeneration, and inflammation lesions could be observed after different treatments, demonstrating the high biocompatibility of IRP NPs.

### 3.6. Biosafety of IRP NPs

**Figure 10.**
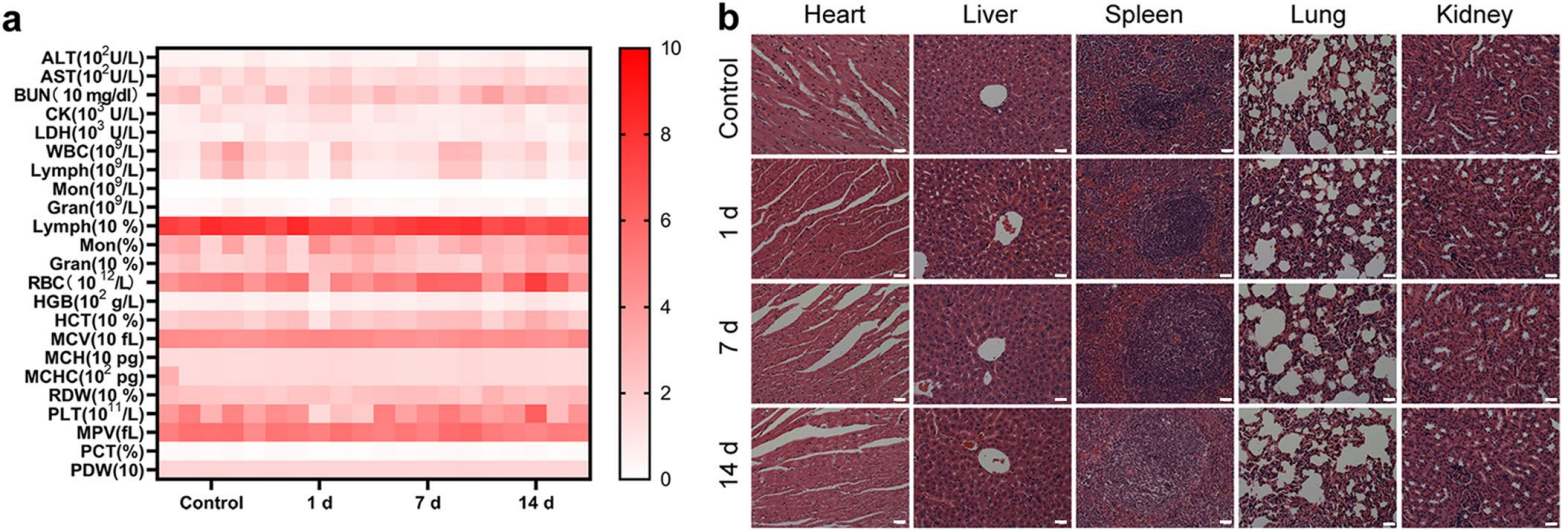
Biosafety of IRP NPs. (a) Biochemical assay and hematology analysis of mice intravenously injected with IRP NPs. The blood samples were collected at pre-determined time points (0, 1, 7, and 14 d) post-injection. (b) H&E staining of tissue sections from major organs after treatments (scale bar: 50 µm).

Finally, we studied the potential toxicity of IRP NPs in healthy mice. After tail vein injection, the main organs and blood samples were collected at a predetermined time for analysis. The main parameters of routine blood tests and biochemistry remained stable without noticeable changes (Figure 10(a)). In addition, no inflammation or tissue damage was observed in the main organs of the mice (Figure 10(b)). All these data prove that IRP NPs have good biocompatibility.

## 4. Conclusions

In conclusion, a novel mitochondria-targeting nanosystem (‘Mito-Bomb’) for ferroptosis-boosted SDT was presented. The nanosystem was selectively localized in the mitochondria of TNBC cells, and ultrasound irradiation resulted in the loss of mitochondrial membrane potential and mitochondrial damage, thereby triggering cell death through a combination of ferroptosis and apoptotic pathways. In addition, the excellent biosafety of IRP NPs makes them more valuable for clinical translation.

## Author contributions

Jianxin Wang: Methodology, Investigation, Software, Writing – original draft; Zhiyu Zhao: Software, Investigation, Visualization; Yan Liu: Software, Methodology; Xinyu Cao: Methodology, Supervision; Fuxin Li: Visualization; Haitao Ran: Resources, Supervision Yang Cao: Conceptualization, Resources, Writing – review & editing; Changjun Wu: Conceptualization, Project administration, Supervision, Resources, Writing – review & editing,

## Supplementary Material

Supplemental Material

## Abbreviations

SDT: sonodynamic therapy
ROS,: reactive oxygen species
PLGA,: poly(lactic‐*co*‐glycolic acid)
HIF-1α,: hypoxia inducible factor-1α
TNBC,: triple-negative breast cancer tumor
LPO,: lipid peroxidation
GPX4,: GSH glutathione peroxidase 4
PAI,: photoacoustic imaging
DiI,: 1,1-dioctadecyl-3,3,3′,3′-tetramethylindocarbocyanine perchlorate
PVA,: polyvinyl alcohol
DCFH-DA,: 2′-7′-dichlorofluorescein diacetate
JC-1,: 5,5′,6,6′-tetrachloro-1,1′,3,3′-tetraethyl-imidacarbocyanine iodide
DAPI: 4,6-diamidino-2-phenylindole
CCK-8,: Cell Counting Kit-8
CH_2_Cl_2_: dichloromethane
DLS,: dynamic light scattering
PBS,: phosphate buffered saline
HPLC,: high-performance liquid chromatography
CLSM,: confocal laser scanning microscope
US,: ultrasound
IP NPs: IP780-PLGA NPs
IRP NPs,: IP780-RSL-3-PLGA NPs
WB,: western blot
TUNEL,: TdT-mediated dUTP nick-end labeling
H&E,: hematoxylin–eosin staining
