# Supplementary material for: ‘Mito-Bomb’: a novel mitochondria-targeting nanosystem for ferroptosis-boosted sonodynamic antitumor therapy
Source: Drug Deliv. 2022 Sep 21;29(1):3111–22. doi: 10.1080/10717544.2022.2126027 (PMC9518294; doi:10.1080/10717544.2022.2126027)
Supplement: Supplemental Material [file IDRD_A_2126027_SM6167.docx]

**Supporting Information**

**“Mito-Bomb”: a novel mitochondria-targeting nanosystem for ferroptosis-boosted sonodynamic anti-tumor therapy**

Jianxin Wang^a^, Zhiyu Zhao^a^, Yan Liu^a^, Xinyu Cao^a^, Fuxin Li^a^, Haitao Ran^b^, Yang Cao^b,*^, Changjun Wu^a,*^

^a^ Department of Ultrasound, The First Affiliated Hospital of Harbin Medical University, Harbin 150001, China

^b^ Chongqing Key Laboratory of Ultrasound Molecular Imaging, Institute of Ultrasound Imaging, Second Affiliated Hospital, Chongqing Medical University, Chongqing 400010, China

***Corresponding authors.**

E-mail addresses: yangcao@cqmu.edu.cn (Y. Cao), bccjw@sohu.com (C. Wu).


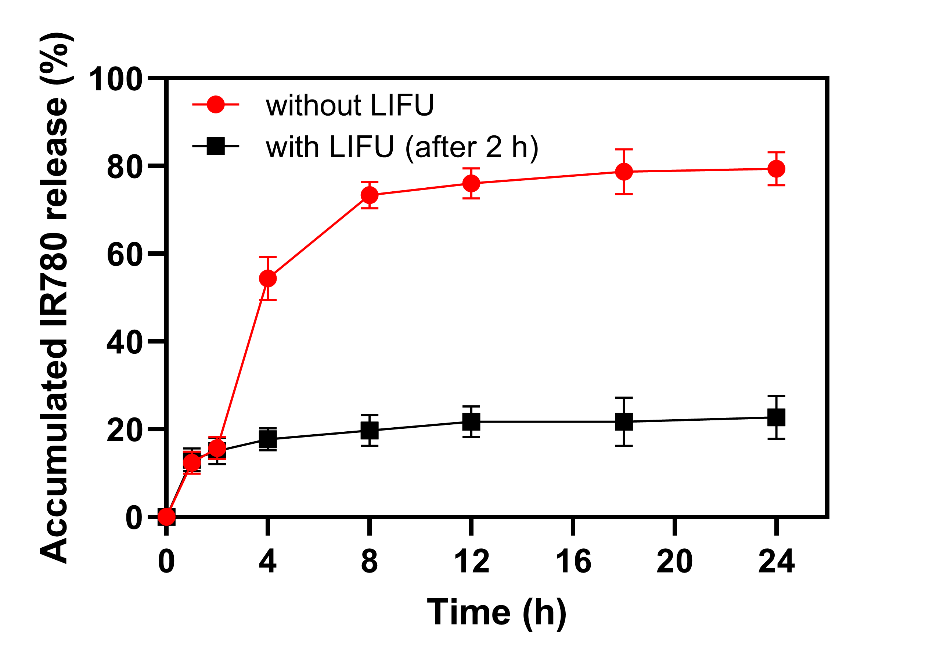


**Figure S1** IR780 release curves from IRP NPs with or without LIFU irradiation.


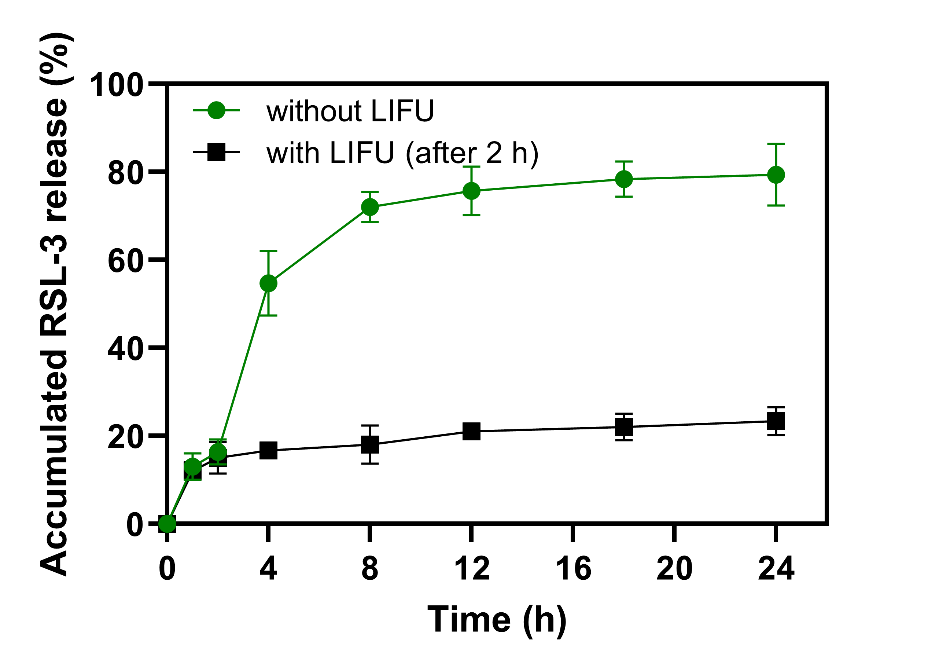


**Figure S2** RSL-3 release curves from IRP NPs with or without LIFU irradiation.


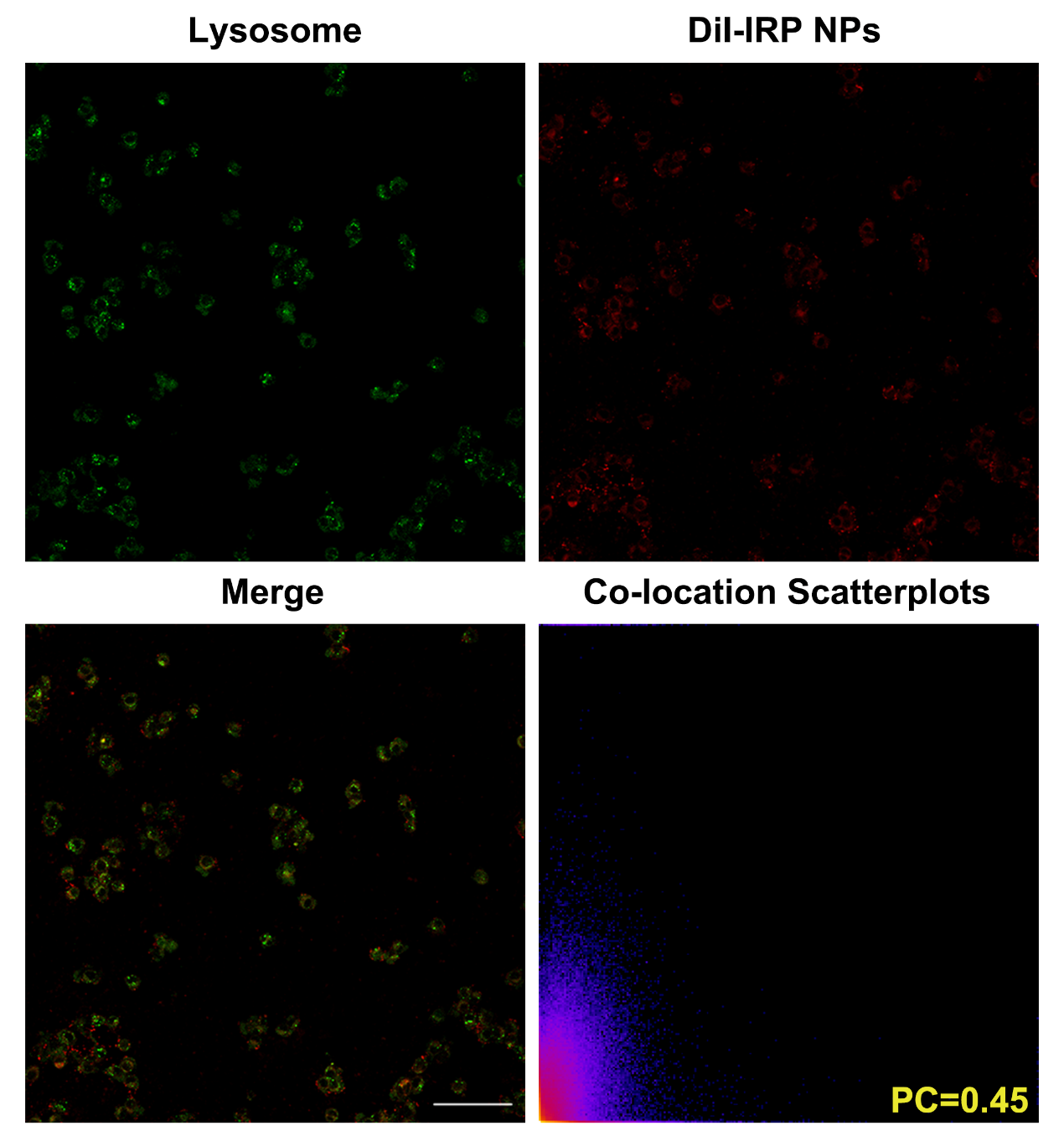


**Figure S3** Lysosome location of DiI-labeled IRP NPs as monitored by LysoTracker (scale bar: 100µm).


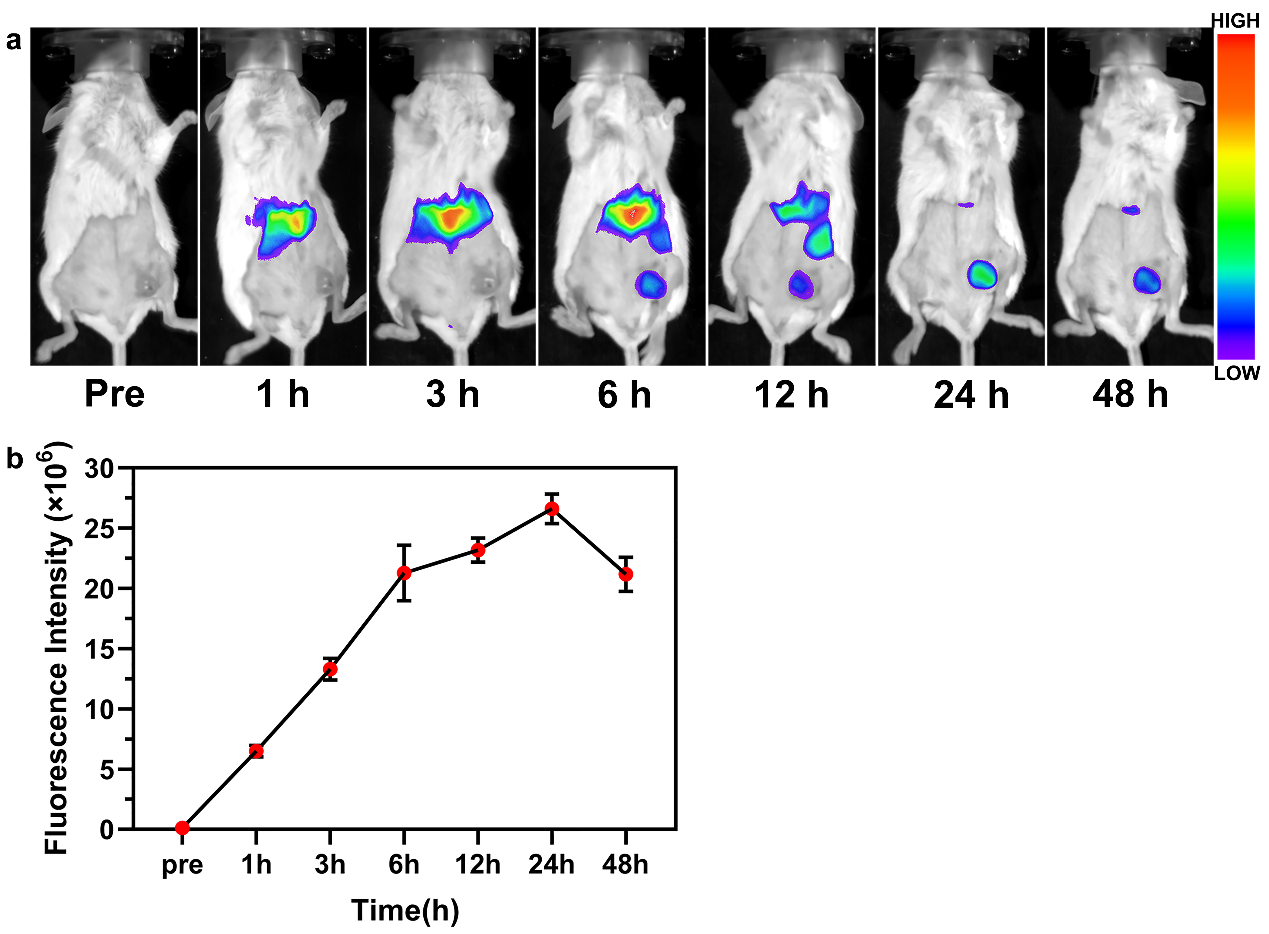


**Figure S4 a** In vivo fluorescence images of tumors in 4T1‐tumor‐bearing mice after injection of IRP NPs at different time points. **b** Changes of fluorescence signal intensities within tumor regions at corresponding time points.
